# Psychology: Objects sticking in memory

**DOI:** 10.1038/s44271-023-00006-z

**Published:** 2023-08-01

**Authors:** Marike Schiffer

**Affiliations:** Communications Psychology, https://www.nature.com/commspsychol

**Keywords:** Cognitive neuroscience, Human behaviour, Learning and memory

## Abstract

Using a vast dataset of object ratings, a new study in *Science Advances* sheds light on the question what makes objects memorable. The answer to what role typicality plays turns out to be complex.

**Figure:**
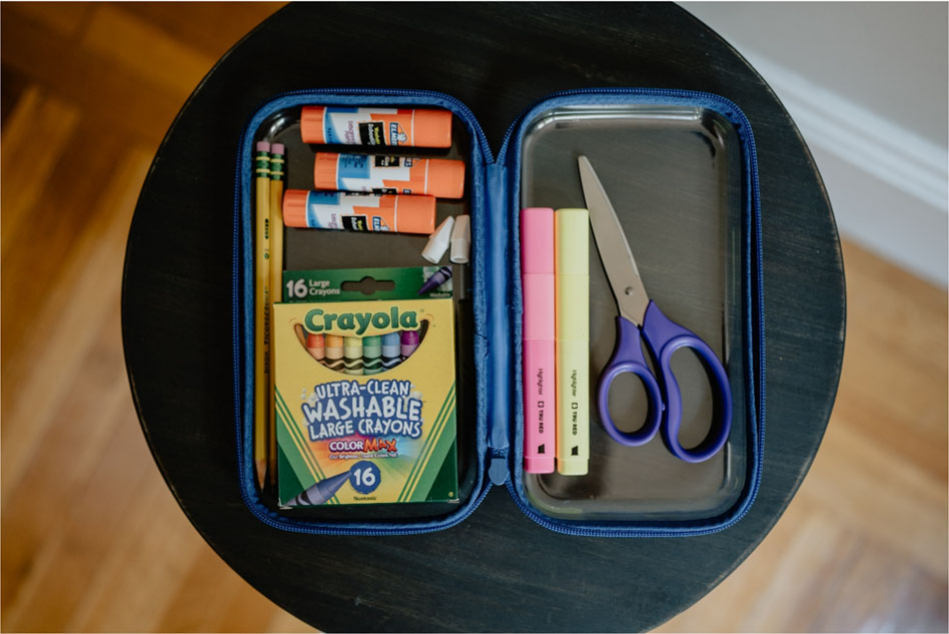
Kelly Sikkema on Unsplash

When a group of people encounter the same range of objects, individuals from that group tend to remember the same objects. But what is the essence of an object that makes it memorable, while others are forgotten?

Max Kramer at the University of Chicago and colleagues used a vast database of images to study this question^1^. The authors were particularly interested in whether there were differences in memorability between conceptual categories of objects (e.g. body parts or parts of a car), what role visual features play, and whether how typical an object is of its category mattered. To this end, they tested the memorability of 26,107 object images for which they had information on these characteristics in a study involving 13,946 participants on the platform MTurk. The authors found that some concepts were more memorable, with body parts being remembered more than car parts. Semantic characteristics of the objects was far more important than visual aspects to how well they were remembered. Finally, the authors uncovered an interesting paradox for prototypicality, i.e. what influence how much an object looks like the typical example of its type has on its memorability. Overall, typical objects were more memorable, however, for many object concepts, the relationship was reversed: less typical objects of that concept were remembered better.

The size of the study and the expanse and complexity of the dataset make this work stand out. And while the study cannot fully explain the differences between object concepts and why they matter for the memorability for particularly typical or atypical examples, it certainly will inspire a lot more work in this domain.

## References

[1] Kramer, M. A. et al. The features underlying the memorability of objects. *Sci. Adv.* **9**, eadd2981 (2023). 37126552 10.1126/sciadv.add2981 PMC10132746

